# Voice Care and Dedication celebrate Ten Years! Celebrate with the ABLV!

**DOI:** 10.1016/S1808-8694(15)30772-2

**Published:** 2015-10-19

**Authors:** Jeferson Sampaio D'Avila

**Affiliations:** Chairman of the Brazilian Academy of Laryngology and Voice Head of the Otorhinolaryngology Program – Federal University of Sergipe Medical School

In 1999, exactly ten years ago, some physicians met with a common concern: laryngeal cancer, which victimized 15 thousand Brazilians per year, eight thousand of those were fatal cases. Meanwhile, our bodies' cries for help regarding the worsening of vocal disorders were being ignored by most people. Having such situation in mind, physicians members of the SBLV (Brazilian Association of Laryngology and Voice) which as of 2004, was called ABLV (Brazilian Academy of Laryngology and Voice), launched the “National Voice Campaign”, a program aimed towards education, information and, especially, care for one's voice – which is a working tool for about 70% of Brazilians. During the ten years the campaign has been on, physicians got to know each other, networked, installed measures and changed the lives of many people. “Our major challenge has always been to make this venture permanent and always alive in our country and in the world”, states Dr. Jeferson Sampaio D'Ávila, current ABLV's Chairman.

In the year the campaign was launched, the SBLV then was chaired by Dr. Nédio Steffen. This physician, with broad experience in Otolaryngology, headed the first Voice Campaign actions which, in that year served more than 40 thousand people all over Brazil. “I consider the Voice Campaign an extremely relevant social undertaking, because it fulfills the very reason for being a doctor: the tireless and continuous pursue of health and well being for the population. In these regards, I feel gratified for having had the opportunity to create it and lead it during its launch as Chairman of the ABLV”, states Steffen.

Dr. Agrício Crespo took office as ABLV Chairman the following year, and was followed by Dr. José Antônio Pinto the year after. Moved, Dr. José Antonio remembers the attention the population paid to the campaign. “In all the campaigns we participated, we were always very much surprised by the interest the population had regarding voice disorders, with large attendance in events, talks, classes and shows, as their attendance during the week we held the exams. As we analyzed a group of almost six thousand individuals, we found voice alterations in 39% of them, including cases of vocal fold cancer”. As he sees it, future challenges are the most concerning ones. “Along these ten years, the ABLV showed its effectiveness in the campaign, which besides having a prophylactic face, it has also shown the population other important aspects, such as professional voice, the voice as a working tool, the early diagnosis of laryngeal cancer and other diseases, and the basic care one should have with one's voice as an important tool of human communication. The economic challenges of a very costly campaign, with the lack of sponsors, makes us have an even greater responsibility in maintaining this campaign which brings about many benefits to the population”, says José Antonio.

Among the many important moments of the Voice Campaign history, one of the most striking one was ABLV's victory as a medical entity. The ABLV struggled and in 2003 achieved, thanks to partnerships with many international entities, the establishment of the “World Voice Day”, officially celebrated all over the world on April 16. I Chaired the ABLV when the National Voice Campaign reached the world. It was amazing to see how it grew in four years only, thanks to the hard work of the former Chairmen who preceded me, that of engaged professionals and population support. I am very proud for having participated in such a campaign that helps so many lives every year”, states Dr. Domingos Tsuji, former Chairman of the ABLV.

Statements from former ABLV Chairmen

1997 to 1998 – Dr. Paulo Pontes (SBLV)

“The Voice Campaign started as an educational movement to explain the value of voice in social communication and its involvement in diseases that can be earlier on detected by its alterations. The results attained in these campaigns show their importance, which caused its transformation into a world-wide movement”.

1998 to 1999 – Dr. Marcos Sarvatt (SBLV)

“My greatest dream is to come to a point in which the Campaign will no longer be necessary and that the obvious may prevail: the population will know how to prevent voice and throat disorders, people will be able to recognize its initial signs and symptoms and find public and private health care facilities to care for them with readiness, kindness and competence”.

1999 2000 - Dr. Nédio Steffen (SBLV)

“I consider the Voice Campaign an extremely relevant social undertaking, because it fulfills the very reason for being a doctor: a continuous and tireless pursue of health and well being for the population”.

2000 to 2001 Dr. Agrício Crespo (SBLV)

“In these 10 years, the National Voice Campaign has established itself as an original and creative tool to divulge how important our voices are in human relations and in signaling health and disease. It has crossed borders and made history in Brazilian Otorhinolaryngology. It is with great and renewed pleasure that I celebrate these 10 years of success with the ABLV”.

2001 to 2002 Dr. José Antônio Pinto (SBLV)

“Along these ten years, the ABLV showed its effectiveness in the campaign, which besides having a prophylactic face, it has also shown the population other important aspects of it, such as professional voice, the voice as a working tool, the early diagnosis of laryngeal cancer and other diseases, and the basic care one should have with one's voice as an important tool of human communication”.

2003 to 2004 Dr. Domingos Tsuji (ABLV)

“I am proud of having participated in a campaign that has helped so many lives all these years”.

2005 to 2006 Dr. Geraldo Druck Sant'Anna (ABLV)

“The greatest importance is in divulging to the population the importance of voice preservation and disease prevention measures. It is also important to stress signs and symptoms that may point to potentially severe diseases. I feel very moved when I see how far we've come, as the selflessness of many”.

2007 to 2008 Dr. Jeferson Sampaio D'Ávila (ABLV)

“All these years have been very important for all of us, especially because we have had increasing numbers of colleagues interested in it. The active participation of the population as a whole, the support from many public and private entities and the Campaign being spread to the world as the World Voice Day is a final proof of how the Brazilian people can be receptive”.

